# Senescence-associated inflammation and inhibition of adipogenesis in subcutaneous fat in Werner syndrome

**DOI:** 10.18632/aging.205078

**Published:** 2023-10-03

**Authors:** Daisuke Sawada, Hisaya Kato, Hiyori Kaneko, Daisuke Kinoshita, Shinichiro Funayama, Takuya Minamizuka, Atsushi Takasaki, Katsushi Igarashi, Masaya Koshizaka, Aki Takada-Watanabe, Rito Nakamura, Kazuto Aono, Ayano Yamaguchi, Naoya Teramoto, Yukari Maeda, Tomohiro Ohno, Aiko Hayashi, Kana Ide, Shintaro Ide, Mayumi Shoji, Takumi Kitamoto, Yusuke Endo, Hideyuki Ogata, Yoshitaka Kubota, Nobuyuki Mitsukawa, Atsushi Iwama, Yasuo Ouchi, Naoya Takayama, Koji Eto, Katsunori Fujii, Tomozumi Takatani, Tadashi Shiohama, Hiromichi Hamada, Yoshiro Maezawa, Koutaro Yokote

**Affiliations:** 1 Department of Endocrinology, Hematology and Gerontology, Chiba University Graduate School of Medicine, Chiba, Japan; 2 Department of Pediatrics, Chiba University Graduate School of Medicine, Chiba, Japan; 3 Division of Diabetes, Metabolism and Endocrinology, Chiba University Hospital, Chiba, Japan; 4 Laboratory of Medical Omics Research, Kazusa DNA Research Institute, Kisarazu, Japan; 5 Department of Omics Medicine, Chiba University Graduate School of Medicine, Chiba, Japan; 6 Department of Plastic, Reconstructive, And Aesthetic Surgery, Chiba University Graduate School of Medicine, Chiba, Japan; 7 Division of Stem Cell and Molecular Medicine, Center for Stem Cell Biology and Regenerative Medicine, The Institute of Medical Science, The University of Tokyo, Tokyo, Japan; 8 Department of Regenerative Medicine, Chiba University Graduate School of Medicine, Chiba, Japan; 9 Department of Clinical Application, Center for iPS Cell Research and Application (CiRA), Kyoto University, Kyoto, Japan; 10 Department of Pediatrics, International University of Welfare and Health School of Medicine, Narita, Japan

**Keywords:** Werner syndrome, premature aging, lipodystrophy, stromal vascular fraction, SASP

## Abstract

Werner syndrome (WS) is a hereditary premature aging disorder characterized by visceral fat accumulation and subcutaneous lipoatrophy, resulting in severe insulin resistance. However, its underlying mechanism remains unclear. In this study, we show that senescence-associated inflammation and suppressed adipogenesis play a role in subcutaneous adipose tissue reduction and dysfunction in WS. Clinical data from four Japanese patients with WS revealed significant associations between the decrease of areas of subcutaneous fat and increased insulin resistance measured by the glucose clamp. Adipose-derived stem cells from the stromal vascular fraction derived from WS subcutaneous adipose tissues (WSVF) showed early replicative senescence and a significant increase in the expression of senescence-associated secretory phenotype (SASP) markers. Additionally, adipogenesis and insulin signaling were suppressed in WSVF, and the expression of adipogenesis suppressor genes and SASP-related genes was increased. Rapamycin, an inhibitor of the mammalian target of rapamycin (mTOR), alleviated premature cellular senescence, rescued the decrease in insulin signaling, and extended the lifespan of WS model of *C. elegans*. To the best of our knowledge, this study is the first to reveal the critical role of cellular senescence in subcutaneous lipoatrophy and severe insulin resistance in WS, highlighting the therapeutic potential of rapamycin for this disease.

## INTRODUCTION

Werner syndrome (WS) is a rare monogenic premature aging disorder caused by *WRN*, a gene that encodes a RecQ-type DNA helicase which is involved in DNA replication and repair [1]. The first symptoms of WS-associated premature aging appear after puberty [2] and include age-related diseases such as diabetes mellitus, dyslipidemia, cardiovascular diseases, and malignant neoplasms [3, 4]. Therefore, research on WS is important as it can provide insights into the pathogenesis and development of treatments not only for WS but also for general age-related diseases [5].

Visceral fat accumulation induces diabetes and other metabolic diseases [6]. Moreover, obesity induces chronic inflammation, leading to insulin resistance [7–9]. However, the role of subcutaneous fat in insulin resistance remains unclear. Lipodystrophy, characterized by the loss of adipose tissues, is often accompanied and aggravated by insulin-resistant diabetes mellitus [10]. WS is characterized by the accumulation of visceral fat and loss of subcutaneous fat (lipodystrophy) in the extremities [11] and is often associated with insulin-resistant diabetes [12], which suggests an association between subcutaneous fat atrophy and insulin resistance. However, the pathogenesis of subcutaneous fat atrophy in WS is not clear. Therefore, this study aimed to reveal the pathogenesis of subcutaneous fat atrophy in the extremities of patients with WS.

## RESULTS

### A decrease in subcutaneous fat is associated with aggravated insulin resistance in patients with WS

**Figure 1:**
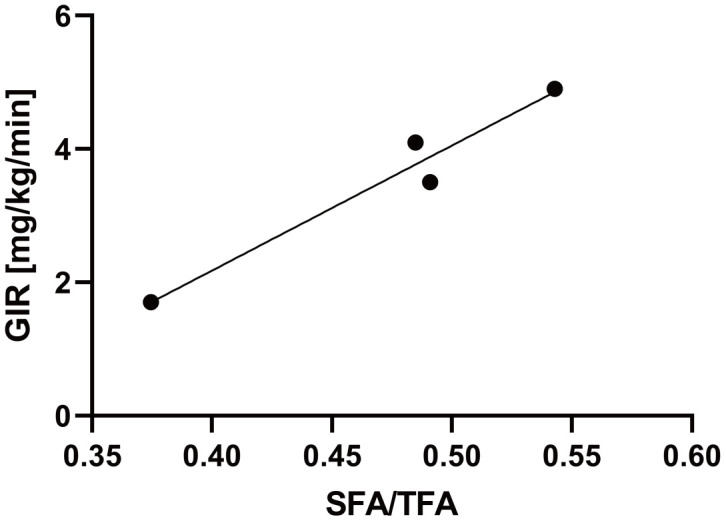
**The SFA/TFA ratio is correlated with the GIR.** Four patients with WS patients were included. The correlation coefficient between the SFA/TFA and GIR; R^2^ = 0.95, *p* = 0.024; for statistical analysis, simple linear regression analysis was performed. WS: Werner syndrome; TFA: total fat area; SFA: subcutaneous fat area; GIR: glucose infusion rate.

In this study, the hyperinsulinemic-euglycemic clamp technique was used to assess insulin resistance in four patients with WS attending our hospital. The characteristics of the four patients are listed in Table 1. Two of the four patients were female, and the median age of the patients was 46 years old (Table 1). The median values of the body mass index (BMI) and skeletal muscle mass index (SMI) were 19.5 and 4.7, respectively, indicating sarcopenia with a relatively small body weight (Table 1). Moreover, we assessed visceral and subcutaneous fat areas using abdominal computed tomography (CT), which showed visceral fat area (VFA) accumulation and a low percentage of the subcutaneous fat area (SFA) (Table 1). The median value of glucose infusion rate (GIR) was 3.8 mg/kg/min, indicating insulin resistance (normal range > 6.0 mg/kg/min; Table 1). Additionally, to investigate the association between lipodystrophy and insulin resistance, we compared subcutaneous fat area/total fat area (SFA/TFA) to the GIR and found a significant positive correlation (R^2^ = 0.95, *p* = 0.024; Figure 1). These results suggest that subcutaneous fat loss in patients with WS may be associated with aggravated insulin resistance.

**Table 1:** Characteristics of the four patients with WS in our study.

**Case**	**Normal range**	**#1**	**#2**	**#3**	**#4**	**Median**
Age [years old]		48	44	44	64	46
Sex		Man	Woman	Man	Woman	
BMI [kg/m^2^]		19.6	20.3	19.4	15.1	19.5
SMI [kg/m^2^]	M: > 6.87 F: > 5.46	4.7	5.7	4.6	2.7	4.7
TFA [cm^2^]		238.0	248.8	329.0	191.3	243.4
VFA [cm^2^]	< 100	108.9	128.3	205.8	97.4	118.6
SFA [cm^2^]		129.2	120.6	123.2	93.9	121.9
V/S ratio		0.84	1.06	1.67	1.04	1.05
S/T ratio		0.54	0.48	0.37	0.49	0.49
GIR [mg/kg/min]	> 6	4.9	4.1	1.7	3.5	3.8

### The stromal vascular fraction of patients with WS exhibits premature cellular senescence with increased expression of senescence-associated inflammatory genes

**Figure 2:**
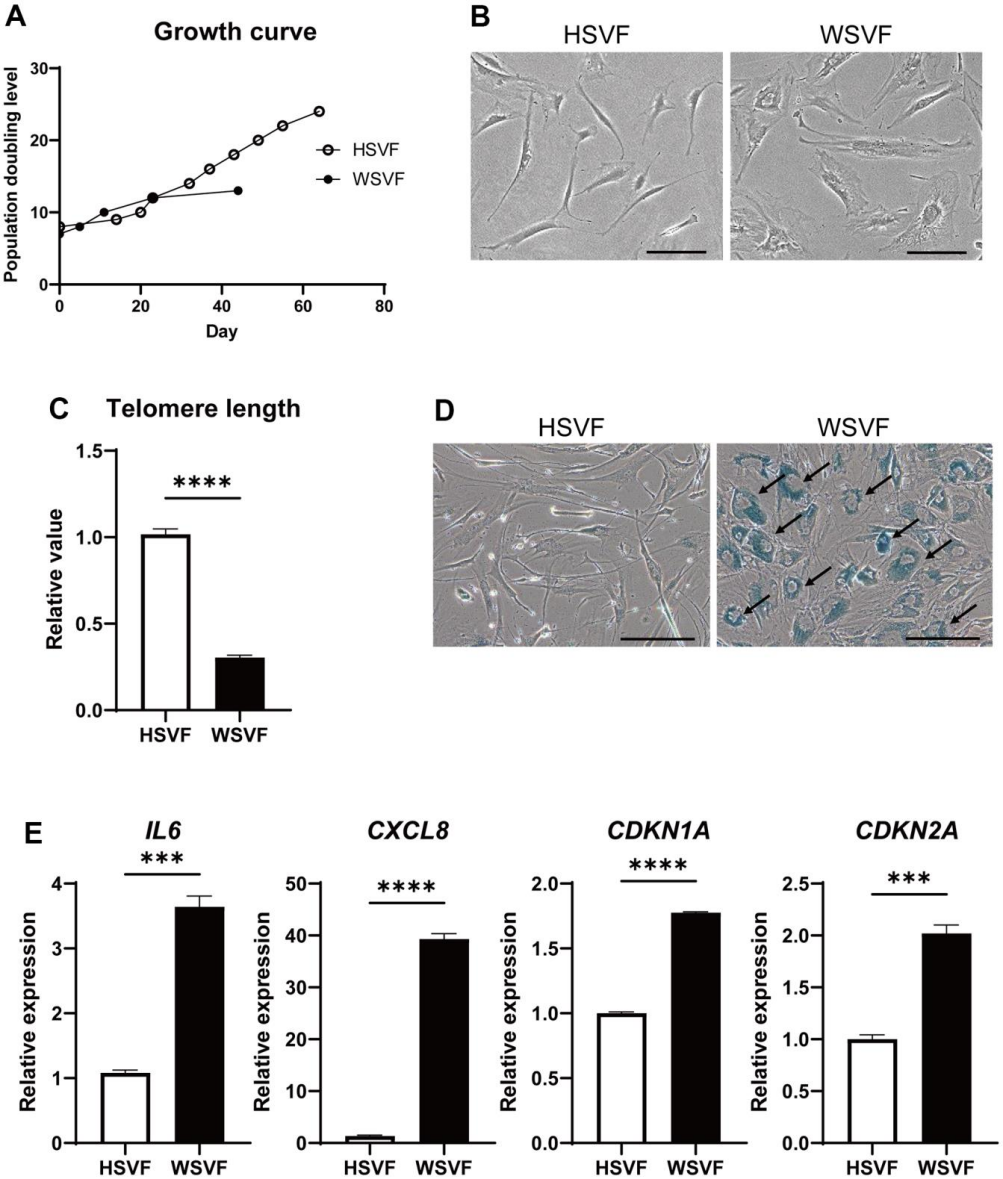
**WSVF exhibits cellular senescence and increased expression levels of inflammatory genes.** (**A**) Growth curves of SVF derived from a healthy individual and a patient with WS. (**B**) Comparison of the morphological features of the SVFs. Scale bar, 300 μm. (**C**) Quantification of the telomere length analyzed by quantitative real-time polymerase chain reaction. Data are presented as means ± S.E.M. of three technical replicates. For statistical analysis, student t-test was performed (****p < 0.0001). (**D**) Representative images of SA-β-gal staining of SVF. Black arrows indicate SA-β-gal-positive cells. Scale bar, 300 μm. (**E**) Quantitative real-time polymerase chain reaction of the relative expression of senescence-related genes. Data are presented as means ± S.E.M. of three technical replicates. For statistical analysis, student t-test was performed (ns, not significant; ***p < 0.001; ****p < 0.0001). WS: Werner syndrome; SVF: stromal vascular fraction; SA-β-gal: senescence-associated β-galactosidase.

Next, we assessed adipose-derived stem cells from the stromal vascular fraction (SVF) obtained from subcutaneous adipose tissues of a patient with WS *in vitro*. The SVF derived from the subcutaneous fat of a 64-year-old healthy individual (HSVF) was compared to that of a 47-year-old patient with WS (WSVF), and both individuals were women. Analysis of the cell growth curve showed that WSVF exhibited premature cell proliferation arrest (Figure 2A). WSVF cells also exhibited senescence-like morphology with enlarged and flattened cell shape at an early passage (population doubling level: PDL 10; Figure 2B). Additionally, quantitative polymerase chain reaction (qPCR) analysis revealed that the telomere length was significantly shortened in WSVF (*p* < 0.0001; Figure 2C). The number of senescence-associated β-galactosidase (SA-β-gal) positive cells was increased in WSVF compared to HSVF (indicated by black arrows, Figure 2D). Furthermore, WSVF showed increased expression levels of senescence-associated inflammatory cytokines, SASP genes, such as *IL6* (*p* < 0.001) and *CXCL8* (*p* < 0.0001). The expression of *CDKN1A* (*p* < 0.0001) and *CDKN2A* (*p* < 0.001), major cyclin-dependent kinase inhibitors, also increased in WSVF compared to HSVF (Figure 2E). These results indicate that the SVF of patients with WS exhibits premature cellular senescence with increased expression levels of SASP genes.

### WSFV exhibits distinct gene expression

**Figure 3:**
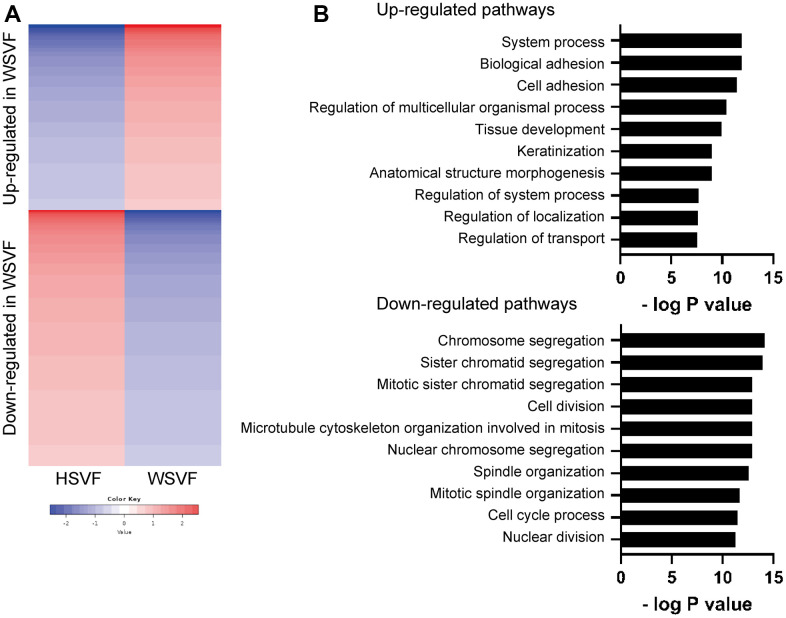
**Transcriptome analysis reveals distinct gene expression in WSVF.** (**A**) k-means clustering of HSVF and WSVF. (**B**) List of the top ten gene ontology terms and corresponding p values related to Figure 3A. Pathway analysis of the top 2000 transcriptome using GO biological process. WS: Werner syndrome; SVF: stromal vascular fraction; HSVF: SVF derived from a healthy individual; WSVF: SVF derived from a patient with WS.

To investigate the gene expression and pathways involved in cellular senescence in WSVF, we performed a transcriptome analysis of WSVF and HSVF. We analyzed the data using k-means clustering and conducted a pathway analysis using gene ontology (GO) biological processes. The top 2000 genes with the largest changes in expression were analyzed; 821 genes were upregulated and 1179 genes were downregulated in WSVF compared to HSVF (Figure 3A, 3B). Pathway analysis using GO biological processes revealed that genes related to cell adhesion and cell structure were upregulated in WSVF whereas those related to chromosome organization and segregation, nuclear division, and cell cycle were downregulated (Figure 3B). These results were consistent with premature cellular senescence.

### Adipogenesis is suppressed in WS

**Figure 4:**
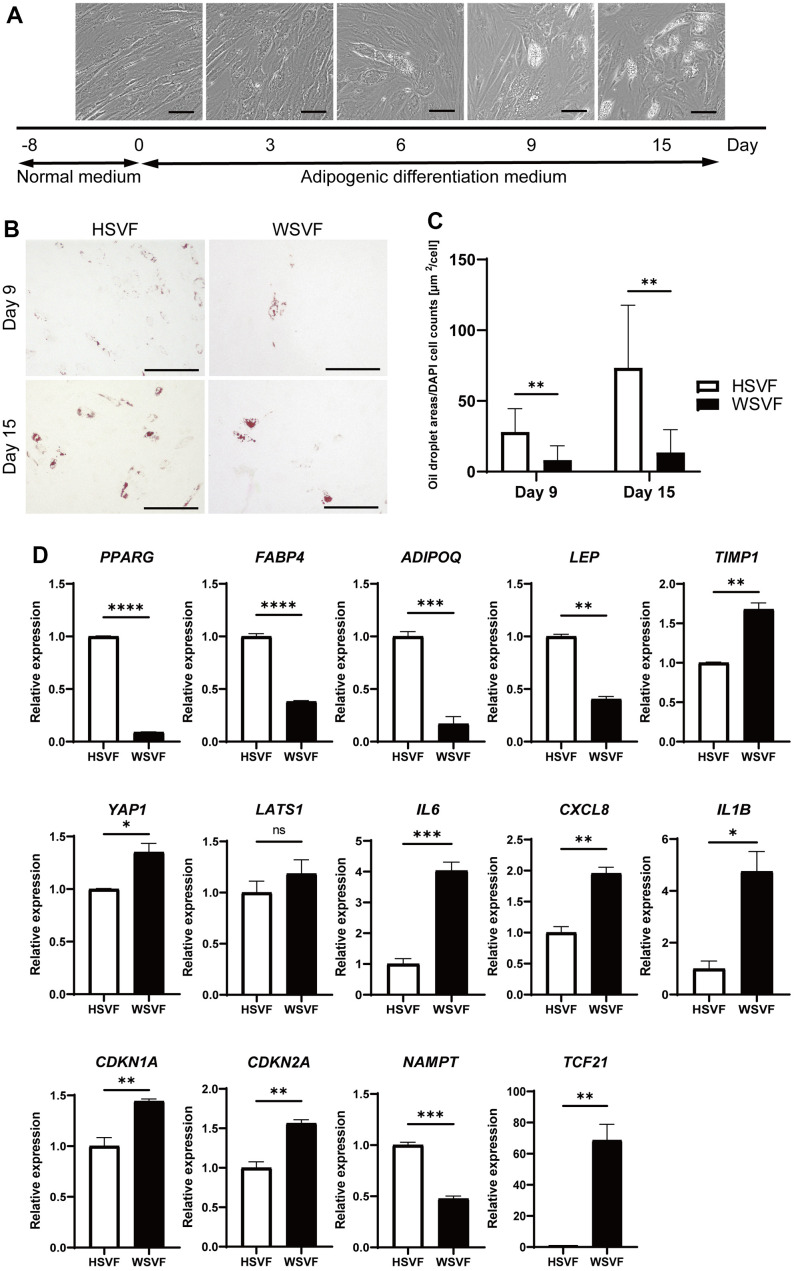
**Adipogenesis is suppressed in WS.** (**A**) Schematic illustration of the adipogenesis experiment. Representative images on days 0, 3, 6, 9, and 15. Scale bar, 100 μm. (**B**) Representative images of Oil Red O staining on days 9 and 15 after adipogenesis in HSVF and WSVF. Scale bar, 300 μm. (**C**) Quantification of the oil droplet area based on DAPI cell counts. Data are presented as means ± S.E.M. from nine different microscopic views. For statistical analysis, student t-test was performed (**p < 0.01). (**D**) Quantitative real-time polymerase chain reaction of the relative gene expression during adipogenesis of three technical replicates. For statistical analysis, student t-test was performed (ns, not significant; *p < 0.05; **p < 0.01; ***p < 0.001). WS: Werner syndrome; SVF: stromal vascular fraction; HSVF: SVF derived from a healthy patient; WSVF: SVF derived from a patient with WS.

We subsequently performed adipogenesis experiments to evaluate the adipogenic potential of WSVF. The protocol for adipogenic differentiation is shown in Figure 4A. During adipogenesis, the number of cells with lipid droplets increased in HSVF (Figure 4A). Oil Red O staining showed that the stained areas were reduced in WSVF on both days 9 and 15 (Figure 4B). Moreover, quantification of the Oil Red O-stained area showed that the number of cells positive for Oil Red O staining was significantly decreased in WSVF compared to that in HSVF on both days 9 and 15, indicating suppressed adipogenesis in WSVF (*p* < 0.01; Figure 4C). During adipogenesis, WSVF exhibited decreased expression levels of *PPARG* (*p* < 0.0001) and *FABP4* (*p* < 0.0001), adipogenesis-related genes, and *ADIPOQ* (*p* < 0.001) and *LEP* (*p* < 0.01) compared to HSVF (Figure 4D). In contrast, WSVF exhibited increased expression levels of *TIMP1* (*p* < 0.01) and *YAP1* (*p* < 0.05), which are suppressors of adipogenesis [13, 14] but decreased expression levels of *NAMPT* (*p* < 0.001), which is a gene related to mitochondrial function, compared to HSVF (Figure 4D). Furthermore, during adipogenesis, the expression levels of the inflammatory molecules SASP, such as *IL6* (p < 0.001), *CXCL8* (*p* < 0.01), and *IL1B* (*p* < 0.05), and those of senescence-related cell cycle regulators, such as *CDKN1A* (*p* < 0.01) and *CDKN2A* (*p* < 0.01), were increased in WSVF compared to HSVF (Figure 4D). Interestingly, *TCF21*, whose expression is usually increased in visceral fat [15, 16], was also increased (*p* < 0.01) in WSVF compared to HSVF (Figure 4D). These results suggest that adipogenesis is suppressed and inflammatory genes are increased in WS.

### Insulin signaling is suppressed in WS

**Figure 5:**
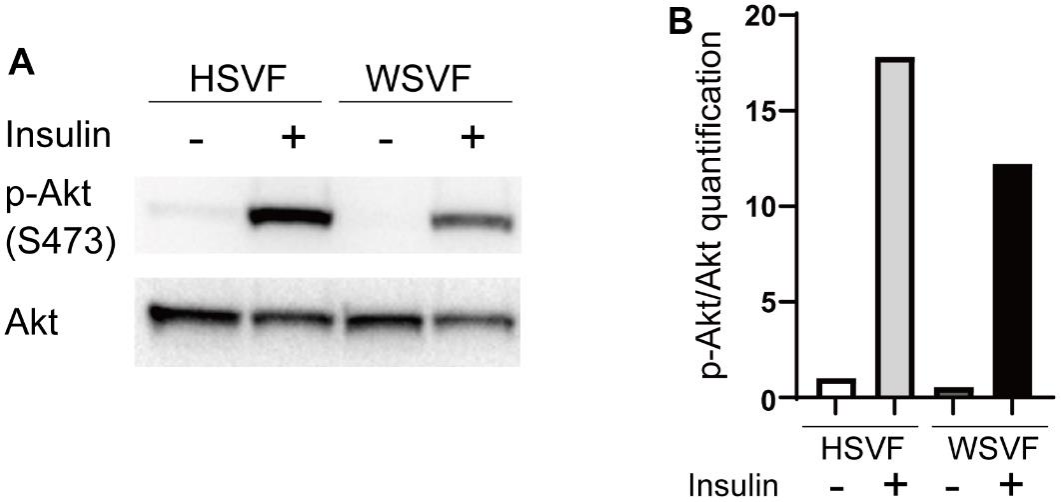
**Insulin signaling is decreased in WS.** (**A**) Western blotting of p-Akt (S473) and Akt for HSVF and WSVF. (**B**) Quantitative analysis of p-Akt/Akt. WS: Werner syndrome; SVF: stromal vascular fraction; HSVF: SVF derived from a healthy patient; WSVF: SVF derived from a patient with WS.

We investigated insulin-related pathways in WSVF using western blotting. After stimulation with insulin, the protein expression levels of p-Akt compared to Akt in WSVF were lower than those in HSVF (Figure 5A). The p-Akt/Akt ratio was reduced to 68.6% in WSVF compared to that in HSVF (Figure 5B). IRS1 and PI3K were decreased in WSVF (Supplementary Figure 3). Moreover, similar results were obtained for WS fibroblasts where the p-Akt/Akt ratio was reduced in WS fibroblasts compared to that in normal fibroblasts (Supplementary Figures 1, 2). These *in vitro* results suggest aggravated insulin resistance in WS.

### Rapamycin ameliorates cellular senescence in SVF and extends the life span of *WRN*-knockout *Caenorhabditis elegans*

**Figure 6:**
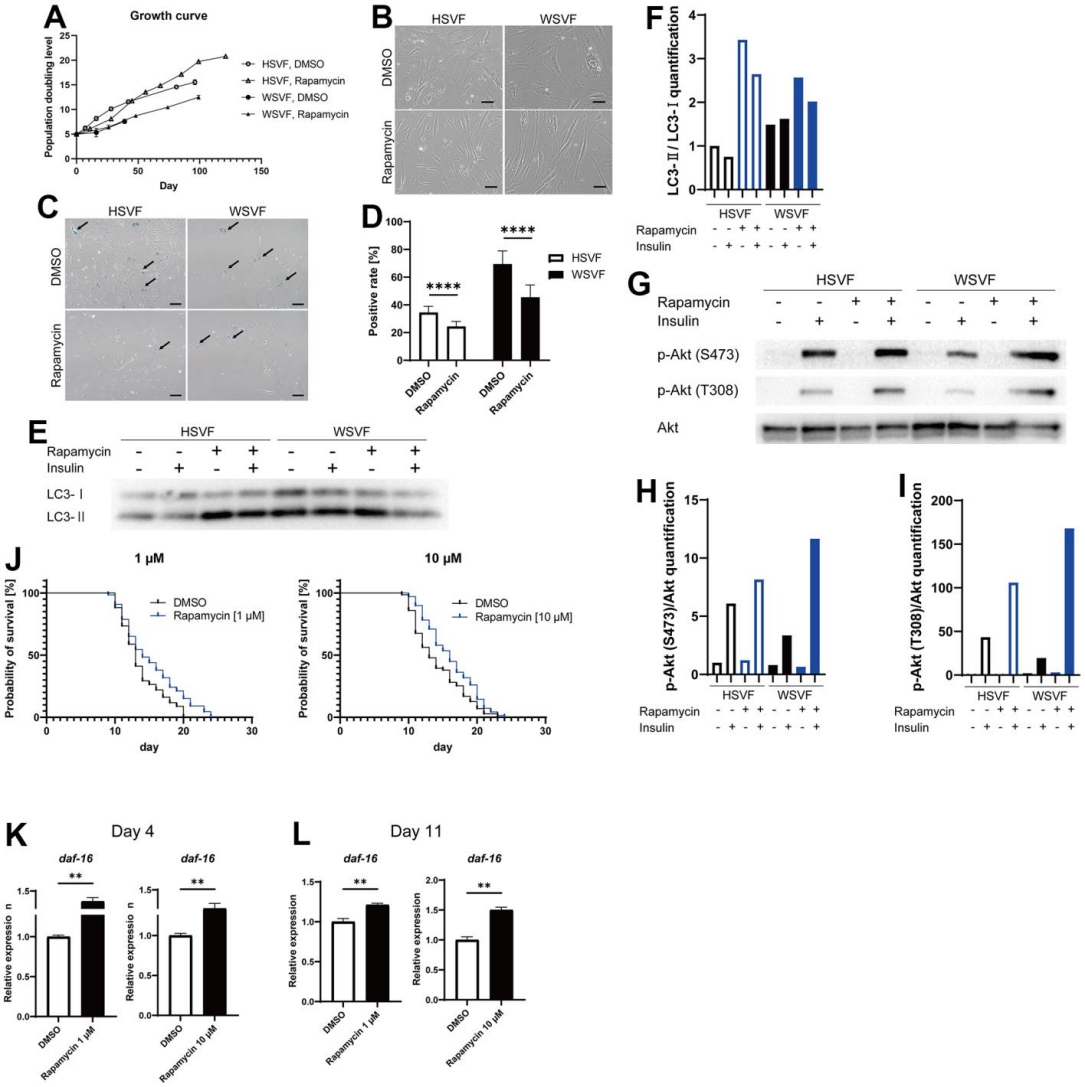
**Rapamycin alleviates cellular senescence in SVF.** (**A**) Growth curves of HSVF and WSVF treated with rapamycin. (**B**) Morphological changes of the SVFs treated with rapamycin. Scale bar, 100 μm. (**C**) Representative images of SA-β-gal staining of the SVFs treated with rapamycin. Black arrows indicate SA-β-gal-positive cells. Scale bar, 100 μm. (**D**) Quantification of SA-β-gal-positive cells. Data are presented as means ± S.E.M. from nine different microscopic views. For statistical analysis, student t-test was performed (****p < 0.0001). (**E**) Western blotting of the protein expression of LC3-I and LC3-II in HSVF and WSVF. (**F**) Quantification of (**E**). (**G**) Western blotting of p-Akt (S473), p-Akt (T308), and Akt in HSVF and WSVF treated with rapamycin. (**H**) Quantitative analysis of p-Akt (S473)/Akt. (**I**) Quantitative analysis of p-Akt (T308)/Akt. (**J**) Survival probability of *WRN*-knockout *C. elegans* (gk99) treated with 1 μM and 10 μM of rapamycin. For statistical analysis, log-rank (Mantel-Cox) test was performed; **p < 0.01 compared with DMSO in 1 μM of rapamycin, *p < 0.05 compared with DMSO in 10 μM of rapamycin. (**K**, **L**) Quantitative real-time polymerase chain reaction of the relative expression of *daf-16* on days 4 and 11 in gk99 treated with 1 μM and 10 μM of rapamycin. Data are presented as means ± S.E.M. of three technical replicates. For statistical analysis, student t-test was performed (**p < 0.01). WS: Werner syndrome; SVF: stromal vascular fraction; HSVF: SVF derived from a healthy patient; WSVF: SVF derived from a patient with WS; SA-β-gal: senescence-associated β-galactosidase; mTORC1: mammalian target of rapamycin complex 1.

Rapamycin extends the lifespan of many species by inhibiting the mTOR pathway [17]. Therefore, we used rapamycin as an agent to inhibit cellular senescence. Treatment with rapamycin prolonged the final PDL attained in both HSVF and WSVF (Figure 6A). Moreover, rapamycin rescued the altered morphology of WSVF from swollen and flattened senescent cells to spindle-shaped cells (Figure 6B). In addition, treatment with rapamycin resulted in a significant decrease in SA-β-gal-positive or senescent cells in both HSVF and WSVF (*p* < 0.0001; Figure 6C, 6D).

To investigate the effect of rapamycin on autophagy, we investigated LC3 using western blotting and found that LC3-II/LC3-I [18] expression levels were increased in WSVF (Figure 6E, 6F), suggesting that autophagosome excessively accumulates in WSVF. Treatment with rapamycin further increased LC3-II/I ratio in WSVF. The mTOR and S6K phosphorylation were decreased with the addition of rapamycin in SVF, confirming the general effect of rapamycin (Supplementary Figure 3). Rapamycin also restored the p-Akt (S473)/Akt ratio from 55.1% to 191.2% and p-Akt (T308)/Akt ratio from 45.0% to 387.2% in WSVF compared to HSVF under stimulation with insulin (Figure 6G–6I). Moreover, similar results were obtained for WS fibroblasts (Supplementary Figure 2). Furthermore, *in vivo,* 1 μM (*p* < 0.01) and 10 μM (*p* < 0.05) of rapamycin significantly prolonged the life span of *WRN*-knockout *C. elegans* (gk99) (Figure 6J). Moreover, the expression of *daf-16*, a gene repressed by mammalian target of rapamycin complex 1 (mTORC1), increased in gk99 treated with 1 μM and 10 μM of rapamycin on days 4 and 11 (*p* < 0.01) (Figure 6K, 6L). These results suggest that rapamycin rescues cellular senescence and insulin resistance in WSVF, and extends the lifespan of the WS model *in vivo*.

## DISCUSSION

The present study revealed for the first time, that a decrease in subcutaneous fat mass to total fat mass ratio was associated with aggravated insulin resistance in patients with WS. We revealed that SVF derived from subcutaneous fat tissues of patients with WS exhibited premature cellular senescence, accompanied by elevated SASP and suppression of adipogenic differentiation *in vitro*. Furthermore, we demonstrated that rapamycin rescues cellular senescence in WSVF and extended the life span of *WRN*-knockout *C. elegans* (gk99) *in vivo*.

Adipose tissue is an insulin-sensitive organ that is important for metabolic homeostasis [19]. Lipodystrophy causes atrophy of the subcutaneous fat in the extremities and is accompanied by severe insulin-resistant diabetes [10]. Patients with WS develop sarcopenic obesity, in which visceral fat accumulates, subcutaneous tissue atrophies, and skeletal muscle loss progresses at a young age [20]. These patients tend to have high blood insulin levels before the onset of diabetes and higher insulin resistance than their peers [12]. Hutchinson-Gilford progeria syndrome, a hereditary premature aging syndrome similar to WS, is also characterized by lipodystrophy in the extremities [21]. Additionally, the general older adult population also exhibits subcutaneous tissue and skeletal muscle loss with age, as well as aggravated insulin resistance [22, 23]. The present study suggests that subcutaneous fat loss, which progresses with age, plays a major role in insulin resistance.

The association between subcutaneous fat atrophy in the extremities and aging is unknown. In the present study, our results suggest that cellular senescence-induced SASP leads to suppressed adipogenesis, ultimately playing a role in subcutaneous fat mass loss. Cellular senescence has been previously demonstrated in fibroblasts derived from patients with WS [24] and in mesenchymal stem cells derived from *WRN*-knockout embryonic stem (ES) cells [25]. In this study, we demonstrated for the first time that cellular senescence also occurs in SVF derived from the subcutaneous fat of patients with WS. Senescent cells secrete inflammatory cytokines and SASPs [26–28], which induce chronic inflammation, promote aging, and contribute to the progression of age-related diseases [29, 30]. Cellular senescence suppresses adipogenesis [31], and SASP acts on adipogenic progenitor cells to inhibit adipogenesis [32], suggesting that WSVF may inhibit its adipogenic differentiation by the autocrine effect of its secreted SASP. Additionally, SVF homogenizes into adipose-derived stem cells by passaging culture [33]; therefore, the cellular senescence of WSVF may reflect aging at the stem cell level. In this study, we observed upregulated expression levels of *YAP1* and *TIMP1*, which are known to suppress adipogenesis [13, 14]. Telomere dysfunction activates YAP and induces inflammation [34]. Moreover, nucleotide excision repair-deficient mice develop adipose loss due to chronic inflammation [35], and *WRN*-deficient ES cells exhibit suppressed adipogenesis [36]. Collectively, these findings suggest that SASP induced by a defective DNA damage response suppresses subcutaneous fat differentiation in the extremities, causing lipodystrophy in WS.

**Figure 7:**
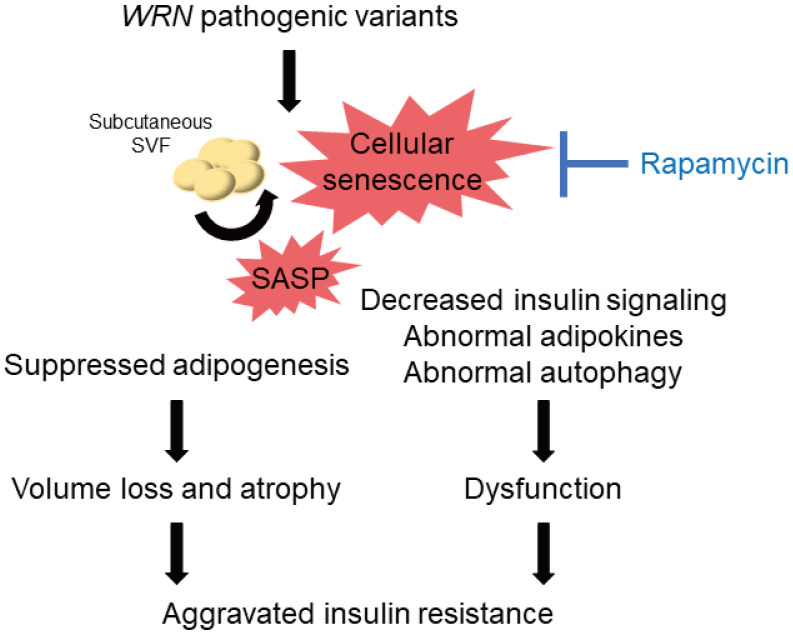
**Schematic illustration of the lipodystrophy and insulin resistance exhibited by patients with WS.** The genomic repair defect caused by pathogenic variants of the *WRN* gene leads to chronic inflammation and cellular senescence, resulting in inhibition of adipogenesis and dysfunction of adipocytes, leading to subcutaneous fat mass and quality loss, which in turn leads to subcutaneous fat atrophy and insulin resistance in patients with WS. WS: Werner syndrome; SVF: stromal vascular fraction; SASP: senescence-associated secretory phenotypes.

A decrease in the quality of the remaining subcutaneous adipocytes might occur in patients with WS. Cellular senescence leads to insulin resistance in adipocytes [37], and suppression of senescent cells accumulated in adipose tissues by blocking TP53 improves insulin resistance [38]. In the present study, we observed reduced insulin signaling in both WF fibroblasts and WSVF, which is consistent with a previous study reporting reduced insulin signaling in WS fibroblasts [39]. We also observed decreased expression of adipokine genes such as leptin and adiponectin. Leptin is decreased in lipoatrophy, and leptin supplementation improves insulin resistance [40]. A recent case report reported the efficacy of leptin supplementation in WS [41]. In addition, subcutaneous fat has been reported to increase with improved glucose tolerance when troglitazone is administered to patients with type 2 diabetes [42]. Pioglitazone also improves insulin resistance, fat distribution, and adipokine abnormalities in WS [43, 44] and Cockayne syndrome, another form of premature aging [45]. Therefore, in addition to the control of subcutaneous fat mass, improvement of the quality or function of subcutaneous adipocytes is important to treat insulin resistance in WS (Figure 7).

The mTOR pathway is one of the pathways involved in the molecular pathogenesis of premature aging [46]. Rapamycin has been reported to extend the lifespan of various organisms by inhibiting the mTOR pathway [17, 47, 48], and its effectiveness has also been demonstrated in *WRN*-deficient human fibroblasts [49]. The previous report also showed that rapamycin treatment reduced the accumulation of DNA damage due to the clearance of damaged proteins in WRN-deficient human fibroblasts [49]. Our results may suggest that autophagosome excessively accumulates in WSVF and that treatment with rapamycin alleviates this state. We also revealed for the first time that rapamycin extends the lifespan of *WRN*-knockout *C. elegans* (gk99), demonstrating its potential therapeutic application in WS. *daf-16* is a gene corresponding to human *FOXO*, which is repressed by mTORC1. *daf-16* is activated by rapamycin which suppresses mTORC1. Activated *daf-16* is involved in life span extension [50]. In this study, the gene expression of *daf-16* was upregulated in gk99 treated with 1 μM and 10 μM of rapamycin on days 4 and 11, supporting that treatment with rapamycin contributed to the prolongation of the lifespan in WS model of *C. elegans*. Additionally, rapamycin not only alleviates senescence but also improves adipogenic differentiation [51, 52] and insulin resistance [53]. Therefore, regulation of the mTOR pathway is a promising therapeutic target for cellular senescence, subcutaneous fat atrophy, and dysfunction in WS (Figure 7).

The Akt-mediated insulin signaling pathway and the mTOR pathway may be depicted as a series of pathways, but they may be complexly related by interactive cross-talk and feedback effects [54–56]. While there is a report that rapamycin extended the life span of *C. elegans* by activating daf-16 [50], some reports indicates that rapamycin increases insulin signaling [57–59], and the increased insulin signaling may assumingly suppress *daf-16* and its orthologs [56]. Moreover, a previous report suggested that rapamycin directly activates lysosomal function independent of mTOR [60]. It is speculated that rapamycin may have multiple points of effect on multiple pathways via mTOR inhibition or in mTOR-independent manner.

This study has several limitations. First, although WS is a rare disease, the number of cases in which insulin resistance was assessed using glucose clamping was limited. Moreover, WSVF was difficult to obtain, which restricted the amount of data and experiments that could be performed. However, only factors with robust changes of 2-fold or more were analyzed using RNA-seq. Future studies using patient-derived iPS-driven differentiated adipocytes are needed to validate our results. Moreover, visceral adipose-derived SVF was not analyzed because of the unavailability of samples. Further studies comparing visceral fat-derived SVF and subcutaneous SVF are needed to reveal the cause of insulin-resistant diabetes in WS to determine the phenotypic differences based on the regions of the adipose tissue.

## MATERIALS AND METHODS

### Clinical patient data and hyperinsulinemic-euglycemic clamp test

Physical examination, fat distribution, and insulin resistance were evaluated in four patients with WS. Physical examination included BMI and SMI. Abdominal CT was used to assess fat distribution, including visceral, and subcutaneous fat areas. The hyperinsulinemic-euglycemic clamp test was used to assess insulin resistance. The insulin infusion rate was maintained at 1.25 mU/kg/min, and the glucose infusion rate was measured.

### Establishment of SVF and cell culture

Subcutaneous adipose tissue was obtained from one healthy individual (a 64-year-old woman) and one patient with WS (a 47-year-old woman). The adipose tissue derived from the patient with WS was transplanted from the abdominal subcutaneous fat to the lower extremities, and the remainder was used for this study. The adipose tissue derived from the healthy individual was used for fat reduction surgery, and the remainder was used in this study. SVF was isolated and established from adipose tissues. Cell culture was performed with DMEM (043-30085, Wako Pure Chemical, Osaka, Japan) supplemented with 10% fetal bovine serum (FBS, 10270106; Gibco, Thermo Fisher Scientific, Waltham, MA, USA) and antibiotic-antimycotic (15240062, Gibco) in humidified 5% CO_2_ air. Collagen-type I-coated cell culture plates (4810-010; AGC TECHNO GLASS Co., Ltd., Shizuoka, Japan) were used. The medium was changed every two days. When sub-confluency was reached, cells were passaged at a 1:4 split ratio until growth arrest, and population doubling level was calculated as previously described [24].

### Quantitative polymerase chain reaction

RNA was extracted and reverse-transcribed as previously described [61]. TaqMan Gene Expression Assays for IL6 (Hs00174131_m1), CXCL8 (Hs00174103_m1), CDKN1A (Hs00355782_m1), CDKN2A (Hs00923894_m1), FABP4 (Hs01086177_m1), CEBPA (Hs00269972_s1), ADIPOQ (Hs00605917_m1), LEP (Hs00174877_m1), TIMP1 (Hs00171558_m1), YAP1 (Hs00902712_g1), LATS1 (Hs01125524_m1), IL1B (Hs01555410_m1), NFKB1 (Hs00765730_m1), NAMPT (Hs00237184_m1), TCF21 (Hs00162646_m1), daf-16 (Ce02422838_m1), GAPDH (Hs02786624_g1), and rps-23 (Ce02465854_g1) were purchased from Applied Biosystems (Thermo Fisher Scientific). Quantification was performed using the Ct method with GAPDH and rps-23 as an internal control. Telomere length analysis was performed by qPCR using SYBR Green PCR Master Mix (Applied Biosystems).

### SA-β-gal staining

The Senescence β-Galactosidase Activity Assay Kit (fluorescence, plate-based; #23833; Cell Signaling Technology, Danvers, MA, USA) was used for SAβgal staining according to the manufacturer’s protocol. Cells were stained overnight at 37° C in a room CO_2_ incubator air. The cells were washed with phosphate-buffered saline (PBS) (−) and stained with Hoechst 33342 (H342; Dojindo, Kumamoto, Japan). Imaging and quantification of stains were performed using a BZ-X700 microscope (Keyence, Osaka, Japan).

### Transcriptomic analysis

For transcriptomic analysis, mRNA was extracted from the SVF at PDL 10. RNA sequencing was performed at the Kazusa DNA Research Institute. The obtained CSV file data were analyzed using iDEP (http://bioinformatics.sdstate.edu/idep/) as previously described [62]. Gene clustering was performed by analyzing the top 2,000 genes with variable expression using k-means.

### Adipose differentiation and Oil Red O staining

SVF was cultured in DMEM (043-30085, Wako) supplemented with 10% FBS (10270106, Gibco) and antibiotic-antimycotic (15240062, Gibco) to full confluency. The day the medium was changed to adipogenic differentiation medium (A10070-01, StemPro^®^ Adipogenesis Differentiation Kit; Gibco) was designated day 0. SVF at PDL 10 was used. Oil Red O staining (Sigma-Aldrich, St. Louis, MO, USA) was performed on days 9 and 15 of cell differentiation. The staining was quantified using a BZ-X700 microscope (Keyence).

### Western blotting

Cultured cells were collected in Laemmli buffer, heated to 95° C, and proteins were extracted. Western blotting was performed according to standard protocols, and images were captured using ChemiDoc (Bio-Rad Laboratories, Hercules, CA, USA). Primary antibodies against GAPDH (D16H11, XP^®^ Rabbit mAb, CST, #5174), phospho-Akt (Ser473, D9E XP^®^ Rabbit mAb, CST, #4060), Akt (Antibody Rabbit, CST, #9272), LC3A/B (Antibody Rabbit, CST, #4108), phospho-Akt (Thr308, C31E5E Rabbit mAb, CST, #2965), IRS-1 (Antibody Rabbit, CST, #2382), PI3 Kinase p85 (19H8, Rabbit mAb, CST, #4257), phospho-mTOR (Ser2448, Antibody Rabbit, CST, #2971), mTOR (Antibody Rabbit, CST, #2972), phospho-p70 S6 Kinase (Thr421/Ser424, Antibody Rabbit, CST, #9204), and p70 S6 Kinase (49D7, Rabbit mAb, CST, #2708) were purchased from Cell Signaling Technology. The primary antibodies were diluted to 1:1000. GAPDH and Ponceau-S staining solutions (BCL-PSS-01, Beacle, Inc., Kyoto, Japan) were used as the internal standards.

The secondary antibody, anti-rabbit IgG, HRP-linked whole antibody donkey (#NA934), was purchased from GE Healthcare (Chicago, IL, USA) and diluted to 1:2500. Band quantification was performed using ImageJ Macro, Band/Peak Quantification Tool (https://dx.doi.org/10.17504/protocols.io.7vghn3w).

### Insulin stimulation experiments

The SVF of PDL 7 was used for the insulin stimulation experiments. Serum starvation was performed for 24 h, followed by insulin stimulation for 15 min. The cells were immediately washed twice with PBS on ice and then collected in Laemmli buffer heated to 95° C.

### Treatment with rapamycin

Rapamycin (100 nM; LC Laboratories, Woburn, MA, USA) with DMSO (Wako Pure Chemicals) as the solvent was added to DMEM (043-30085, Wako Pure Chemicals) supplemented with 10% FBS (10270106, Gibco) and antibiotic-antimycotic (15240062, Gibco). The control was a medium supplemented with DMSO diluted to the equivalent concentration. The medium was changed every two days. Cells were subjected to SAβgal staining and RNA analysis using the methods described above.

### Life span of *C. elegans* treated with rapamycin

*WRN*-knockout *C. elegans*, wrn-1 (gk99), was provided as a gift from Dr. Bohr (Biomedical Research Center, Lab. of Molecular Gerontology, National Institute of Aging, Baltimore, MD, USA) [63]. Gk99 was maintained at 23° C as previously described [64].

Age-synchronized nematodes were prepared as previously described [65]. Nematodes were placed on nematode growth media (NGM) plates seeded with *Escherichia coli* OP50 previously described protocols [50]. The day of hatching was set as day 1, and 100 μM 5-fluoro-2′-deoxyuridine (FudR) was added on days 3 and 4, which corresponded to the L4 stage to suppress reproductive function. Rapamycin (LC Laboratories) dissolved in DMSO (Wako Pure Chemicals) was added to the nematode culture medium at final concentrations of 1 μM and 10 μM, and nematodes were reared from day 1, according to previously described protocols [50]. The control plates contained an equivalent concentration of DMSO. The probability of survival of approximately 60 nematodes in a rapamycin-supplemented medium was compared to that of 60 nematodes in the equivalent DMSO-supplemented medium.

## Supplementary Material

Supplementary Figures
