# Supplementary material for: Senescence-associated inflammation and inhibition of adipogenesis in subcutaneous fat in Werner syndrome
Source: Aging (Albany NY). 2023 Oct 3;15(19):9948–64. doi: 10.18632/aging.205078 (PMC10599740; doi:10.18632/aging.205078)
Supplement: Supplementary Figures [file aging-15-205078-s001.pdf]

SUPPLEMENTARY FIGURES

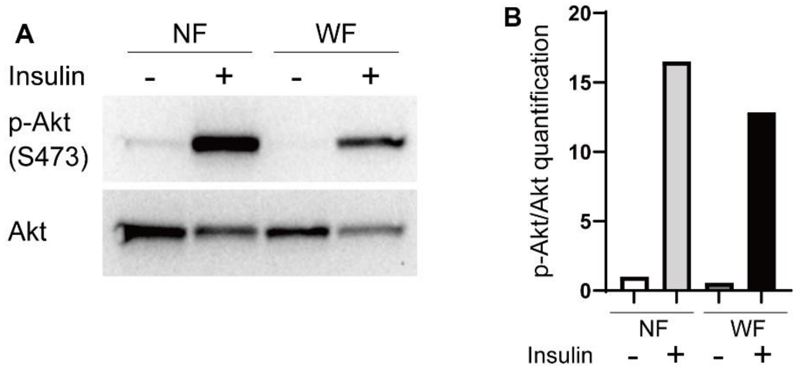

Supplementary Figure 1. Insulin signaling is decreased in WF. (**A**) Western blotting of p-Akt (S473) and Akt in NF and WF. (**B**) Quantitative analysis of p-Akt/Akt. NF: fibroblasts from a normal individual; WF: fibroblasts from a patient with WS; WS: Werner syndrome.

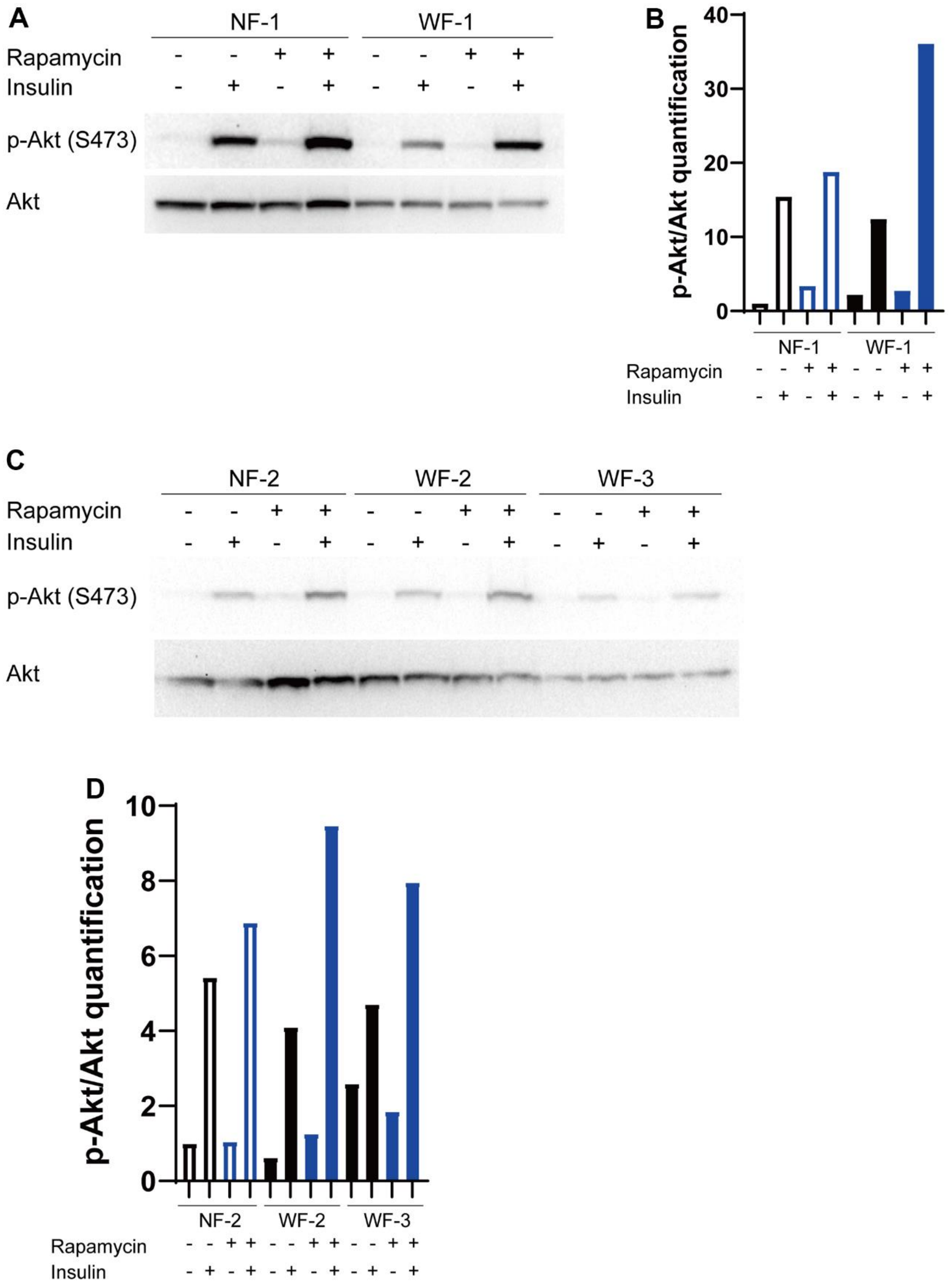

Supplementary Figure 2. Rapamycin alleviates decreased insulin signaling in WF. (**A**) Western blotting of p-Akt (S473) and Akt in NF-1 and WF-1 treated with rapamycin. (**B**) Quantitative analysis of p-Akt/Akt in NF-1 and WF-1. (**C**) Western blotting of p-Akt (S473) and Akt in NF-2, WF-2, and WF-3 treated with rapamycin. (**D**) Quantitative analysis of p-Akt/Akt in NF-2, WF-2, and WF-3. NF: fibroblasts from a normal individual; WF: fibroblasts from a patient with WS; WS: Werner syndrome.

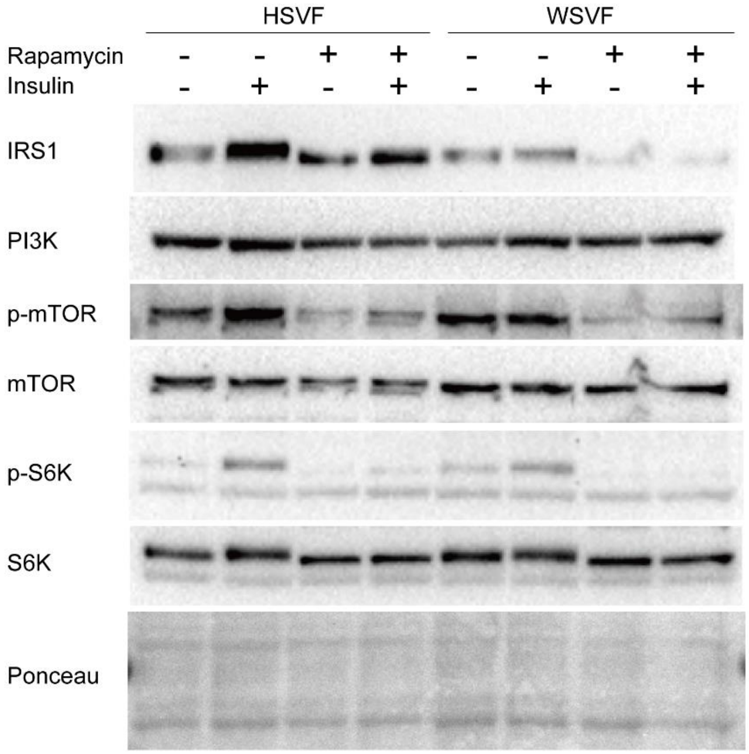

Supplementary Figure 3. Western blotting of IRS1, PI3K, p-mTOR, mTOR, p-S6K, and S6K for HSVF and WSVF. WS: Werner syndrome; SVF: stromal vascular fraction; HSVF: SVF derived from a healthy patient; WSVF: SVF derived from a patient with WS.
